# Complete mitochondrial genome of the monogonont rotifer *Brachionus rotundiformis* (Rotifera, Brachionidae)

**DOI:** 10.1080/23802359.2016.1202743

**Published:** 2017-01-18

**Authors:** Hui-Su Kim, Dae-Sik Hwang, Hee-Jin Kim, Yoshitaka Sakakura, Atsushi Hagiwara, Jae-Seong Lee

**Affiliations:** a Department of Biological Science, College of Science, Sungkyunkwan University, Suwon, South Korea;; b Graduate School of Fisheries and Environmental Sciences, Nagasaki University, Nagasaki, Japan

**Keywords:** *Brachionus rotundiformis*, complete mitochondrial genome, Monogonont rotifer

## Abstract

The two complete mitochondrial genomes were sequenced from the monogonont rotifer *Brachionus rotundiformis*. The genome sequences were 10,268 bp and 11,703 bp in size, and the gene order and contents were identical with those of *B. koreanus* but were different in tRNA-Cys with *B. plicatilis* mitochondrial genomes. Of the 12 protein-coding genes (PCGs), five genes (*ND1, ATP6, ND5, CO3, ND3*) had incomplete stop codons. Furthermore, the start codon of *ND4* and *CO3* gene was ATA, while the start codon of other PCGs was ATG. The base composition of *B. rotundiformis* mitogenome shows an anti-G bias (12.05% and 10.24%) on the second and third position of the PCGs, respectively.

More than 30 species are described in the genus *Brachionus* (https://en.wikipedia.org/wiki/Brachionus). Of these, 15 species were deciphered for their phylogenetic placement through DNA taxonomy using *CO1* and *ITS1* genes in the *Brachionus plicatilis* species complex (Mills et al. 2016). However, to date, only a few complete mitochondrial genomes have been published in the genus *Brachionus* sp. (Suga et al. 2008 for *B. plicatilis*; Hwang et al. 2014 for *B. koreanus*). *Brachionus rotundiformis* (SS type) is a tropical euryhaline species (Hagiwara et al. 1995) and one of valuable food sources for rearing fish larvae with small mouth gape (Yúfera et al. 1997). The analysis of *B. rotundiformis* mitochondrial genome is important to identify laboratory stocks. In this paper, we report two complete mitochondrial genomes of the monogonont rotifer *B. rotundiformis* to better understand the phylogenetic placement and within the genus *Brachionus*.

The specimens were collected on Java Island, Indonesia in 1986 (kindly provided by Prof. Kazutsugu Hirayama, Nagasaki University, Japan) and maintained at the Laboratory of Professor Atsushi Hagiwara, Nagasaki University in Japan. The type was deposited in the ichthyological collection of the aculty of Fisheries, Nagasaki University (FFNU) under the accession no. FFNU-Rot-0001. We sequenced the whole genome of the rotifer *B. rotundiformis* from whole body genomic DNA of *B. rotundiformis* with paired-end libraries (300 bp, 500 bp, 800 bp) and mate-pair libraries (5 kb, 10 kb) using the Illumina HiSeq 2000 platform (GenomeAnalyzer, Illumina, San Diego, CA). *De novo* assembly was conducted by the ALLPATHS-LG release 44849 (http://www.broadinstitute.org/software/allpaths-lg/blog/). Sequenced reads with a phred score below 30 were removed. Of the assembled 2,390 *B. rotundiformis* scaffolds, two scaffolds were matched with the mitochondrial DNAs of *B. koreanus* (GenBank Nos. KC603851, KC603850). As a result, two mitochondrial genomes were obtained with full length.

The complete mitochondrial genomes of *B. rotundiformis* were 10,268 bp (mitochondrial DNA I; GenBank no. KX364936) and 11,703 bp (mitochondrial DNA II; GenBank no. KX364937). The direction of protein-coding genes (PGCs) was identical with those of *B. koreanus* of the genus *Brachionus* including the presence of nearly identical non-coding region (Identities: 3957/3964) (Suga et al. 2008; Hwang et al. 2013). Between the two species (*B. rotundiformis*, *B. koreanus*), the similarities of amino acids and nucleotides of 12 PCGs were 80.55% (96.12% for *CO1* and 92.61% for *Cytb*) and 73.52% (82.21% for *CO1* and 81.75% for *Cytb*), respectively. Of the 12 PCGs, five genes (*ND1, ATP6, ND5, CO3, ND3*) had incomplete stop codons as shown in *B. plicatilis* (Suga et al. 2008) and *B. koreanus* (Hwang et al. 2014). Particularly, in *B. rotundiformis*, there is an anti-G bias (12.05% and 10.24%) at the second and third position of codons. The start codon of *ND4* and *CO3* gene was ATA, while the start codon of other PGCs genes was ATG. The mitochondrial genome base composition of 12 PCGs was 24.45% for A, 45.40% for T, 13.77% for G and 16.38% for C. The A + T base composition (69.85%) was higher than G + C (43.65%).

The placement of *B. rotundiformis* in the genus *Brachionus* with 11 mitochondrial DNA genes was shown in Figure 1. *B. rotundiformis* clustered closely to *B. plicatilis* (L type) (Mills et al. 2016). Interestingly, in the genus *Brachionus*, *tRNA-Cys* was translocated between *tRNA-Arg* and *tRNA-Ile* in *B. plicatilis*, while *tRNA-Cys* of other *Brachionus* species was conserved in order in the mitochondrial genome. This indicates that the rearrangement of tRNAs is likely occurring in sporadic manner in the genus *Brachionus*.

**Figure 1.**
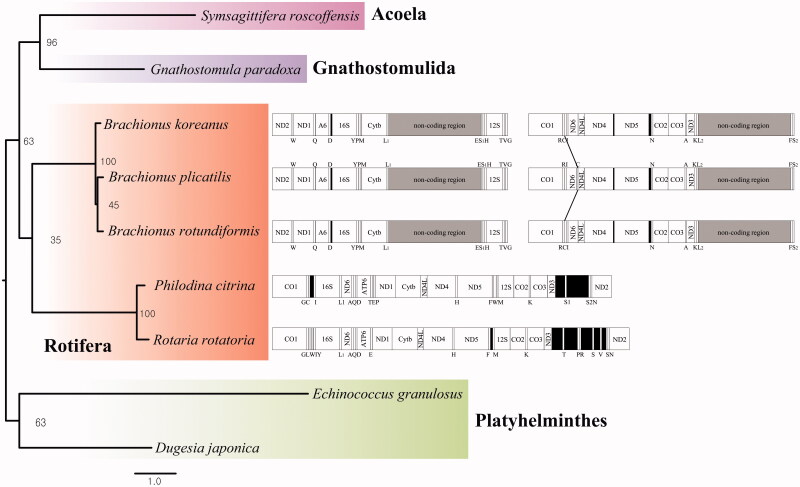
Phylogenetic analysis. We conducted a comparison of the 11 mitochondrial DNA genes except for *ND4L* gene of Acoela, Gnathostomulida, Platyhelminthes, and Rotifera. The 11 mitochondrial DNA genes were aligned by ClustalW. Maximum likelihood (ML) analysis was performed by Raxml 8.2.8 (http://sco.h-its.org/exelixis/software.html) with GTR + Gamma + I nucleotide substitution model. The rapid bootstrap analysis was conducted with 10,000 replications with 48 threads running in parallel. The complete mitochondrial genomes were shown in parallel with a phylogenetic tree. The line on the mitochondrial genome indicates a translocation of tRNAs. The Platyhelminthes served as outgroup.
